# A conjoint experiment of three placebo rectal products used with receptive anal sex: results from MTN‐035

**DOI:** 10.1002/jia2.26219

**Published:** 2024-03-17

**Authors:** José Bauermeister, Willey Lin, Ryan Tingler, Albert Liu, Suwat Chariyalertsak, Craig Hoesley, Pedro Gonzales, Ken Ho, Noel Kayange, Thesla Palanee Phillips, Sherri Johnson, Elizabeth Brown, Jillian Zemanek, Cindy E. Jacobson, Gustavo F. Doncel, Jeanna Piper, the MTN‐035 Protocol Team for the Microbicide Trials Network

**Affiliations:** ^1^ University of Pennsylvania Philadelphia Pennsylvania USA; ^2^ Bridge HIV at the San Francisco Department of Public Health San Francisco California USA; ^3^ Research Institute for Health Sciences, Chiang Mai University Chiang Mai Thailand; ^4^ University of Alabama at Birmingham Birmingham Alabama USA; ^5^ IMPACTA, Asociación Civil Impacta Salud y Educación, San Miguel CRS Lima Perú; ^6^ University of Pittsburgh Pittsburgh Pennsylvania USA; ^7^ Blantyre CRS, Johns Hopkins University Research Project Blantyre Malawi; ^8^ Wits Reproductive Health and HIV Institute Johannesburg South Africa; ^9^ FHI 360 Durham North Carolina USA; ^10^ Statistical Center for HIV/AIDS Research & Prevention, Fred Hutchinson Cancer Research Center Seattle Washington USA; ^11^ CONRAD, Eastern Virginia Medical School Norfolk Virginia USA; ^12^ Division of AIDS United States National Institute of Health, National Institute of Allergy and Infectious Diseases Bethesda Maryland USA

**Keywords:** acceptability, HIV prevention, men who have sex with men, microbicides, PrEP, transgender

## Abstract

**Introduction:**

End‐user perspectives are vital to the design of new biomedical HIV prevention products. Conjoint analysis can support the integration of end‐user perspectives by examining their preferences of potential pre‐exposure prophylaxis (PrEP) products. The Microbicides Trial Network (MTN) 035 protocol examined three placebo rectal dosage forms (insert, enema and suppository) that could deliver PrEP prior to receptive anal sex (RAS).

**Methods:**

Between April 2019 and July 2020, we enrolled 217 HIV‐negative, cisgender men who have sex with men (MSM; *n* = 172; 79.3%) and transgender people (*n* = 47; 20.7%) ages 18–35 into a randomized cross‐over trial across Malawi, Peru, South Africa, Thailand and the United States. Participants used each product prior to RAS over 4‐week periods. Participants completed a conjoint experiment where they selected between random profiles using seven features (dosage form, timing of use before sex, side effects, duration of protection, effectiveness, frequency of use and need for a prescription).

**Results:**

Effectiveness was the strongest determinant of choice (30.4%), followed by modality (18.0%), potential side effects (17.2%), frequency of use (10.8%), duration of protection (10.4%), timing of use before sex (7.4%) and need for a prescription (5.9%). Relative utility scores indicated that the most desirable combination of attributes was a product with 95% efficacy, used 30 minutes before sex, offering a 3‐ to 5‐day protection window, used weekly, having no side effects, in the form of an enema and available over‐the‐counter.

**Conclusions:**

Choice in next‐generation PrEP products is highly desired by MSM and transgender people, as no one‐size‐fits‐all approach satisfies all the preferences. MTN‐035 participants weighed product features differently, recognizing the need for diverse, behaviourally congruent biomedical options that fit the needs of intended end‐users.

## 1 INTRODUCTION

Clinical advances in the success of pre‐exposure prophylaxis (PrEP) have resulted in a greater number of PrEP modalities (e.g. daily oral, long‐acting injectable, microbicide vaginal ring) from which individuals can choose. The availability of PrEP modalities, alongside a suite of products currently in clinical development (e.g. rings, films, implants, vaccines and rectal microbicides [RMs]) [1, 2], has reinforced the need to understand how individuals make decisions regarding their preferred HIV prevention strategies. Preferences in HIV prevention strategies may also vary based on diverse socio‐cultural contexts, including the availability of PrEP modalities and healthcare infrastructure across regions [3−5].

RMs, defined as topical biomedical products being developed to reduce the risk of HIV and other sexually transmitted infections (STIs) for use prior to or after sex [1, 6, 7], are one set of products currently under study. RMs could provide a prevention alternative for people seeking a pericoital prevention modality, as opposed to a systemic PrEP modality [7−9]. If found to be safe and effective, RMs could be designed to deliver HIV/STI prevention drugs and be behaviourally congruent with users’ lifestyles and contexts [1, 10, 11, 12]. Enemas, suppositories and fast‐dissolving inserts [13−16] could be viable rectal delivery modalities given their high hypothetical acceptability as potential RM vehicles [13, 17, 18]; however, the hypothetical nature of these findings may not translate into real‐world acceptability and use. Moreover, prior RM acceptability research has suggested that future acceptability and use may vary across contexts [7, 9, 19, 20]. To address this gap in the literature, the Microbicide Trials Network (MTN) designed the MTN‐035 (DESIRE; Developing and Evaluating Short‐Acting Innovations for Rectal Use) protocol to assess participants’ experiences with these three modalities prior to receptive anal intercourse (RAI) across five different countries (Malawi, Peru, South Africa, Thailand and the United States of America).

Conjoint analysis can be used to identify the importance and salience of product characteristics (i.e. features; e.g. effectiveness, mode of delivery, potential side effects) across levels (i.e. attributes; e.g. 50% effective; 80% effective; 95% effective) that may affect intended users’ uptake of these products [7, 21]. This manuscript describes MTN‐035 participants’ choice trade‐offs after having the opportunity to use each of three rectal placebo modalities (i.e. enema, suppository, fast‐dissolving insert) prior to receptive anal sex (RAS). Our study has three objectives. First, we used a conjoint analysis method to estimate participants’ given importance of key features often considered in RM acceptability studies (e.g. effectiveness, mode of delivery, timing of use before sex, duration of protection, side effects). Consistent with prior research [6, 7, 10, 11, 19, 20, 22], we hypothesized that higher HIV prevention effectiveness and the absence of side effects would be the two most important features reported by participants. Second, we examined participants’ preferred attributes across the features studied. We hypothesized that participants would prefer an enema compared to other delivery modalities given its high behavioural prevalence among men who have sex with men (MSM) and transgender women [13, 23]. We also hypothesized that participants would prefer a highly effective and enduring product that might be available over the counter for use immediately before sex and without side effects. Finally, we examined participants’ preferred product combination based on the included relative utility scores calculated in the conjoint analysis. We hypothesized that the preferred product combination would be a highly effective enema that could be used right before engaging in RAS and that would not have negative side effects.

## 2 METHODS

### 2.1 Sample

Two hundred and seventeen HIV‐negative transgender men, transgender women and cisgender MSM between the ages of 18 and 35 were recruited into the trial [24]. Data collection took place between April 2019 and July 2020 in Malawi (Blantyre), Peru (Lima), South Africa (Johannesburg), Thailand (Chiang Mai) and the United States (Pittsburgh, Pennsylvania; Birmingham, Alabama; and San Francisco, California).

Participants were recruited from a variety of sources, including outpatient clinics, universities, community‐based locations, online websites and social networking applications. In addition, participants were also referred to the study from other local research projects and other health and social service providers. The study was reviewed and approved by the Institutional Review Boards/Ethics Committees at all participating institutions. This study was submitted to clinicaltrials.gov on 14 September 2018, assigned number NCT03671239.

### 2.2 Eligibility and enrolment

Participants were screened for eligibility prior to enrolling in the study. Inclusion criteria included: (1) men (cis or transgender) and transgender women between 18 and 35 years old; (2) ability and willingness to provide written informed consent in local language; (3) HIV‐1/2 seronegative at Screening and Enrolment; (4) ability and willingness to provide adequate locator information; (5) availability to return for all study visits and willingness to comply with study participation requirements; (6) in general good health at Screening and Enrolment; (7) a reported history of consensual RAI at least three times in the past 3 months and expectation to maintain at least that frequency of RAI during study participation; (8) willingness to not take part in other research studies involving drugs, medical devices, genital or rectal products, or vaccines for the duration of study participation; (9) for individuals who could get pregnant (transgender men with a female reproductive system), a negative pregnancy test at Screening and Enrolment; and (10) for individuals who could get pregnant, use of an effective method of contraception for at least 30 days (inclusive) prior to Enrolment, and intention to use an effective method for the duration of study participation.

Enrolled participants provided written informed consent. Participants returned to the clinic within a 45‐day screening window where they completed administrative, behavioural, clinical and laboratory procedures. Additionally, clinical results or treatments for urinary tract infections, genital/reproductive tract infections, sexually transmitted infections (UTIs/RTIs/STIs) or other findings were provided as clinically indicated at all visits. At all clinic visits, participants were also dispensed male condoms and lubricant.

### 2.3 Study procedures

**Figure 1:**
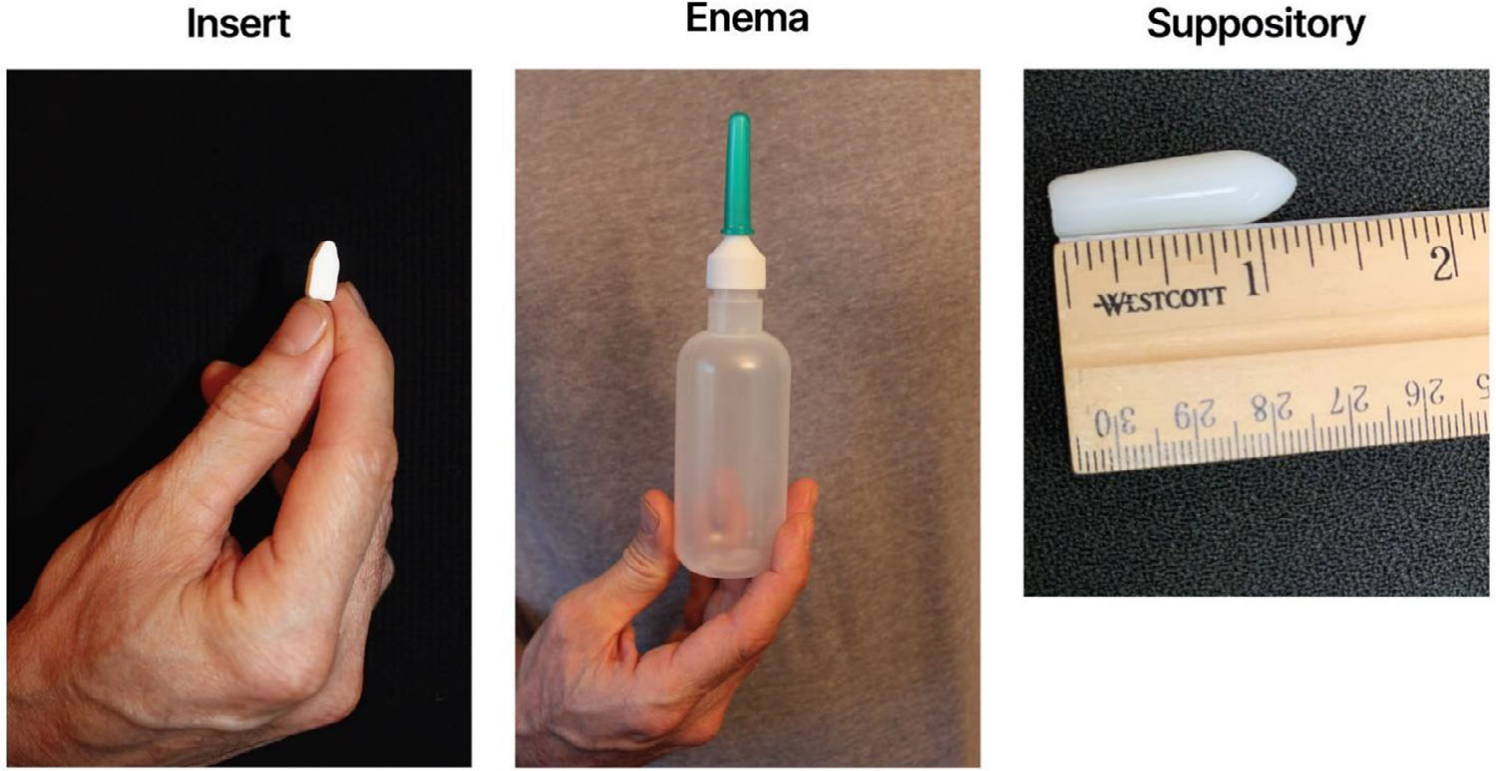
Illustrations of placebo study products used in the Microbicides Trial Network 035 trial.

Consented and enrolled participants were randomized into one of six sequences, each varying the order in which participants used the study placebo products, with a 1‐week wash‐out period between each 4‐week product use period. Each participant received placebo inserts, placebo suppositories and placebo (water) enema bottles for pericoital rectal administration (see Figure 1). The rectal suppository was approximately 3−3.8 cm (1.2−1.5 inches) long and 2 grams in weight. The placebo rectal suppository consists of a Witepsol® H5 (IOI Oleochemical) base and contains 15% diglyceride and not more than 1% monoglyceride content. The placebo rectal insert provided by CONRAD is formulated into white to off‐white uncoated solid dosage forms in a bullet shape. The insert contains the following inactive excipients: isomalt, xylitol, sodium CMC, povidone, hydroxypropyl methylcellulose, poloxamer 188, sodium stearyl fumarate and magnesium stearate. The insert was 1.5 cm (0.6 inches) long, 0.7 cm (0.28 inches) wide, 0.6 cm (0.23 inches) in height and approximately 500 mg in weight.

The products were administered in order of the assigned sequence and prior to each respective product use period. Participants were instructed to use one dose of the assigned study product between 30 minutes and 3 hours prior to RAI, following their usual pre‐RAI practices, and not to use more than one product dose in 24 hours. If a participant did not engage in RAI in a given week, they were asked to insert a dose of the product in the absence of RAI. Participants self‐administered the first dose of each product in the clinic to ensure correct administration.

At Visit 2, participants were provided with their first rectal product for period 1, based on their assigned sequence. For Visits 3, 5 and 7, participants returned to the clinic for the product use end visits. After an approximately 7‐day washout period following study product use periods, participants returned to the clinic to complete Visits 4 and 6. At these visits, participants completed study procedures, including pharyngeal, urine, blood, pelvic (individuals with a vagina or neovagina) and anorectal tests. Additionally, participants self‐administered one dose of the product they were dispensed and collected the remaining product in their sequence to use for the next 4 weeks during periods 2 and 3; they were also given product use instructions.

During their final visit (Visit 8), participants completed a conjoint analysis assessment. Visit 8 served as the follow‐up safety contact and termination visit where participants completed study procedures as well as received clinical results or treatment for UTIs/RTIs/STIs or other findings.

### 2.4 Measures

#### 2.4.1 Socio‐demographic characteristics

Participants completed a baseline computer‐assisted self‐interview where they reported socio‐demographic characteristics, including age, sex assigned at birth and current gender, race/ethnicity and tribal affiliation for African countries (see Table 1).

**Table 1:** Descriptive statistics of participants’ baseline demographic characteristics

	All sites (*N* = 217)	Birmingham (*N* = 33)	Pittsburgh (*N* = 33)	San Francisco (*N* = 30)	Blantyre (*N* = 31)	Chiang Mai (*N* = 30)	Johannesburg (*N* = 30)	Lima (*N* = 30)
Age (years), M (SD)	24.9 (4.7)	25.7 (5.1)	25.5 (4.8)	28.6 (3.9)	24.6 (4.6)	23.3 (3.3)	21.9 (3.0)	24.7 (4.7)
Sex assigned at birth, *N* (%)								
Male	214 (99%)	31 (94%)	32 (97%)	30 (100%)	31 (100%)	30 (100%)	30 (100%)	30 (100%)
Female	3 (1%)	2 (6%)	1 (3%)	0 (0%)	0 (0%)	0 (0%)	0 (0%)	0 (0%)
Gender								
Men	173 (80%)	28 (85%)	28 (85%)	27 (90%)	19 (61%)	22 (73%)	28 (93%)	21 (70%)
Women	2 (1%)	0 (0%)	0 (0%)	1 (3%)	0 (0%)	0 (0%)	0 (0%)	1 (3%)
Transgender men	2 (1%)	1 (3%)	1 (3%)	0 (0%)	0 (0%)	0 (0%)	0 (0%)	0 (0%)
Transgender women	19 (9%)	2 (6%)	0 (0%)	0 (0%)	0 (0%)	8 (27%)	2 (7%)	7 (23%)
Gender non‐conforming/variant	5 (2%)	0 (0%)	3 (9%)	1 (3%)	0 (0%)	0 (0%)	0 (0%)	1 (3%)
Other gender	10 (5%)	0 (0%)	0 (0%)	1 (3%)	9 (29%)	0 (0%)	0 (0%)	0 (0%)
Multiple genders	6 (3%)	2 (6%)	1 (3%)	0 (0%)	3 (10%)	0 (0%)	0 (0%)	0 (0%)
Latinx ethnicity (U.S. sites)	13 (6%)	2 (6%)	3 (9%)	8 (27%)	0 (0%)	0 (0%)	0 (0%)	0 (0%)
Race (U.S. sites)/ethnic group or tribe (African sites)								
Asian	8 (4%)	0 (0%)	2 (6%)	6 (20%)	0 (0%)	0 (0%)	0 (0%)	0 (0%)
Black or African American	16 (7%)	13 (39%)	1 (3%)	2 (7%)	0 (0%)	0 (0%)	0 (0%)	0 (0%)
Native Hawaiian or Other Pacific Islander	1 (1%)	1 (3%)	0 (%)	0 (0%)	0 (0%)	0 (0%)	0 (0%)	0 (0%)
White	66 (30%)	19 (58%)	28 (85%)	19 (63%)	0 (0%)	0 (0%)	0 (0%)	0 (0%)
Multiple races	33 (15%)	0 (0%)	2 (6%)	1 (3%)	0 (0%)	0 (0%)	0 (0%)	30 (100%)
Thai	30 (14%)	0 (0%)	0 (0%)	0 (0%)	0 (0%)	30 (100%)	0 (0%)	0 (0%)
Xhosa	5 (2%)	0 (0%)	0 (0%)	0 (0%)	0 (0%)	0 (0%)	5 (17%)	0 (0%)
Zulu	14 (6%)	0 (0%)	0 (0%)	0 (0%)	0 (0%)	0 (0%)	14 (47%)	0 (0%)
Other African tribe	38 (18%)	0 (0%)	0 (0%)	0 (0%)	31 (100%)	0 (0%)	7 (23%)	0 (0%)
Other	6 (3%)	0 (0%)	0 (0%)	2 (7%)	0 (0%)	0 (0%)	4 (13%)	0 (0%)
Pre‐exposure prophylaxis use								
Currently on PrEP	66 (30%)	13 (39%)	16 (49%)	25 (83%)	0 (0%)	6 (20.0%)	6 (20%)	0 (0%)
Previously on PrEP	17 (8%)	2 (6%)	3 (9%)	3 (10%)	0 (0%)	0 (0%)	5 (17%)	4 (13%)
Never taken PrEP	134 (62%)	18 (55%)	14 (42%)	2 (7%)	31 (100%)	24 (80.0%)	19 (63%)	26 (87%)

#### 2.4.2 Conjoint experiment

Each participant was randomized to complete an online conjoint experiment consisting of 12 scenarios. In each scenario, participants were asked to choose between two potential products with distinct attributes. The features and attributes included in this study were: mode of delivery (attributes: enema, suppository, insert), timing of use before sex (attributes: right before sex, 30 minutes before sex, 1−2 hours before sex, more than 2 hours before sex), side effects (attributes: no side effects, some nausea, some gas, some diarrhoea), duration of protection (attributes: less than 6 hours, 6−24 hours, 1−2 days, 3−5 days), effectiveness (attributes: 50%, 65%, 80%, 95%), frequency of use (attributes: every day, every other day, once a week) and need for a prescription (prescription needed, available over the counter).

### 2.5 Data analytic strategy

We used IBM SPSS, version 27, to compute descriptive statistics (e.g. percentages, means and standard deviations) to characterize the sample's baseline characteristics. In consideration of biological differences among participants and their limited small sample size, the three participants assigned female sex at birth (*n* = 3, of whom two identified as transgender men and one identified as non‐binary) were excluded from subsequent aggregate analyses. For the conjoint analyses, we used Qualtrics’ Conjoint Analysis Software Tool to estimate the relative importance of each feature. Greater scores in feature importance would suggest that it carries greater weight during participants’ decision‐making (see Table 2). We then also examined the preference share for the attributes within each feature. In exploratory analyses, we examined whether there were regional differences in participants’ preference shares (see Table 3). After adjusting for multiple comparisons to reduce Type I errors [25], we found no differences across preference shares across regions. Greater scores in preference share indicate that the attribute would be desirable when seeking to combine attributes across features (e.g. optimal product bundle). Finally, we estimated the relative utility value, where greater scores indicated the extent to which an attribute enhances a product bundle by being included.

**Table 2:** Conjoint analysis metrics from participants who completed Visit 8

Feature	Attribute	Importance	Preference share	Relative utility
Delivery	Insert	18	26%	−3
	Suppository		20%	−7.5
	Enema		54%	10.5
Timing before sex	Right before sex	7.4	34%	2.1
	30 minutes before sex		27%	2.1
	1−2 hours before sex		25%	1
	More than 2 hours before sex		13%	−5.3
Side effects	No side effects	17.2	59%	10.3
	Some nausea		7%	−6.9
	Some diarrhoea		11%	−5.8
	Some gas		23%	2.4
Duration of protection	Less than 6 hours	10.4	19%	−5.9
	6−24 hours		16%	−0.4
	Between 1−2 days		27%	1.9
	3−5 days		39%	4.4
Effectiveness	50%	30.4	9%	−14.3
	65%		4%	−7.1
	80%		14%	5.3
	95%		73%	16.1
Frequency of use	Every day	10.8	22%	−5.5
	Every other day		22%	0.2
	Once a week		56%	5.3
Prescription needed	Prescription only	5.9	27%	−2.9
	Over the counter		73%	2.9

**Table 3:** Means and standard deviations of participants’ preference shares by site

		Pittsburgh	San Francisco	Birmingham	Lima	Blantyre	Chiang Mai	Johannesburg
		(*N* = 31)	(*N* = 30)	(*N* = 27)	(*N* = 19)	(*N* = 27)	(*N* = 30)	(*N* = 30)
**Feature**	**Attribute**							
Delivery	Insert	.23 (.28)	.25 (.28)	.18 (.28)	.23 (.34)	.31 (.34)	.40 (.38)	.24 (.31)
	Suppository	.15 (.22)	.26 (.30)	.19 (.26)	.09 (.13)	.20 (.28)	.23 (.27)	.23 (.29)
	Enema	.62 (.38)	.49 (.39)	.63 (.40)	.68 (.42)	.49(.41)	.37 (.40)	.54 (.43)
Timing before sex	Right before sex	.35 (.21)	.42 (.18)	.42 (.21)	.31 (.25)	.29 (.19)	.31 (.24)	.28 (.20)
	30 minutes before sex	.26 (.09)	.26 (.07)	.24 (.07)	.27 (.13)	.32 (.11)	.27 (.09)	.30 (.07)
	1−2 hours before sex	.26 (.13)	.21 (.09)	.23 (.11)	.24 (.10)	.27 (.11)	.28 (.16)	.29 (.13)
	More than 2 hours before sex	.13 (.10)	.11 (.08)	.11 (.09)	.19 (.17)	.12 (.11)	.14 (.10)	.14 (.12)
Side effects	No side effects	.65 (.32)	.71 (.23)	.54 (.34)	.71 (.30)	.48 (.35)	.53 (.37)	.55 (.36)
	Some nausea	.06 (.09)	.05 (.06)	.05 (.09)	.06 (.15)	.08 (.11)	.06 (.07)	.10 (.16)
	Some diarrhoea	.03 (.05)	.03 (.04)	.11 (.17)	.14 (.23)	.22 (.28)	.09 (.15)	.20 (.28)
	Some gas	.26 (.25)	.21 (.20)	.30 (.26)	.09 (.11)	.23 (.26)	.32 (.30)	.15 (.16)
Duration of protection	Less than 6 hours	.11 (.16)	.11 (.19)	.13 (.16)	.16 (.20)	.31 (.31)	.21 (.25)	.31 (.28)
	6−24 hours	.15 (.05)	.15 (.04)	.16 (.05)	.16 (.03)	.16 (.06)	.16 (.06)	.16 (.06)
	Between 1−2 days	.25 (.20)	.30 (.18)	.32 (.18)	.33 (.18)	.24 (.20)	.23 (.17)	.22 (.19)
	3−5 days	.50 (.23)	.44 (.22)	.39 (.25)	.34 (.22)	.30 (.26)	.39 (.30)	.31 (.29)
Effectiveness	50%	.01 (.04)	.005 (.02)	.03 (.07)	.07 (.11)	.16 (.25)	.10 (.20)	.24 (.34)
	65%	.01 (.02)	.02 (.07)	.04 (.08)	.10 (.14)	.09 (.15)	.03 (.06)	.04 (.05)
	80%	.05 (.09)	.07 (.15)	.16 (.20)	.18 (.16)	.23 (.19)	.13 (.14)	.18 (.18)
	95%	.93 (.15)	.91 (.22)	.77 (.22)	.66 (.34)	.53 (.36)	.74 (.31)	.54 (.36)
Frequency of use	Every day	.12 (.21)	.11 (.17)	.16 (.16)	.26 (.25)	.46 (.31)	.17 (.23)	.31 (.26)
	Every other day	.15 (.10)	.18 (.10)	.23 (.14)	.23 (.12)	.25 (.11)	.22 (.12)	.28 (.14)
	Once a week	.73 (.28)	.71 (.22)	.61 (.27)	.51 (.32)	.28 (.31)	.61 (.30)	.42 (.31)
Prescription needed	Prescription only	.76 (.20)	.78 (.18)	.75 (.16)	.73 (.23)	.64 (.25)	.78 (.19)	.68 (.22)
	Over the counter	.24 (.20)	.22 (.18)	.25 (.16)	.27 (.23)	.36 (.25)	.22 (.19)	.32 (.22)

## 3 RESULTS

### 3.1 Sample characteristics

The mean age of the 217 enrolled participants was 24.9 years (SD = 4.7), ranging from 18 to 35 years old. Most of the sample reported having a male sex assigned at birth (*n* = 214; 99%). Twenty percent of the sample identified as a gender minority. The racial, ethnic and tribal affiliation of participants across the study sites is noted in Table 1. Participants’ history of PrEP use in the sample also varied by region (*X* ^2^_(df = 12)_ = 91.04; *p*<0.001), with participants in San Francisco being more likely to be currently on PrEP than counterparts in other regions.

### 3.2 Conjoint experiment

#### 3.2.1 Feature importance

As noted in Table 2, effectiveness (30.4%) was the strongest determinant of choice, followed by product dosage form (18.0%), and side effects (17.2%). Participants placed less importance on a product's frequency of use (10.8%), duration of protection (10.4%), timing of use before sex (7.4%) and need for a prescription (5.9%).

#### 3.2.2 Preference share

A product with 95% effectiveness carried much of the preference share, followed by 80% effectiveness (14%), 50% effectiveness (9%) and 65% effectiveness (4%). The enema (54%) carried most of the preference share, followed by the insert (26%) and suppository (20%). Most participants desired a product that would not have any side effects (59%), followed by some experiences of gassiness (23%), diarrhoea (11%) or nausea (7%). When considering the time needed to wait after using the product, participants’ preference share diminished with greater waiting time before having sex: right before sex (34%), 30 minutes before sex (27%), 1−2 hours before sex (25%) or more than 2 hours before sex (13%). Participants’ most preferred duration of protection was 3−5 days (39%), followed by 1−2 days (27%), less than 6 hours (19%) and 6−24 hours (16%). Participants noted a preference for a product that would need to be used on a weekly basis (56%), followed by either every day (22%) or every other day (22%). An over‐the‐counter option carried most of the preference share (73%) when compared to a product requiring a prescription (27%).

#### 3.2.3 Relative utility

The relative utility of a product's effectiveness was highest at 95% (16.1), followed by 80% effectiveness (5.3). A product with 65% effectiveness (−7.1) or 50% effectiveness (−14.3) was perceived as reducing the overall value of the product. Participants assigned a positive value to the enema (+10.5) and were more willing to accept the insert (−3) when compared to the suppository (−7.5) in creating an optimal product bundle. In examining the relative utility for side effects, participants preferred a combination product that would not have any side effects (10.3) or where they might experience some gassiness (2.4), noting less relative utility for a product that would cause some diarrhoea (−5.8) or nausea (−6.9).

Participants assigned comparable utility to a product that could be used right before sex (2.1) or 30 minutes before sex (2.1), with less relative utility if they had to wait 1−2 hours before sex [1] or more than 2 hours before sex (−5.3) after using the product. The relative utility of a product's duration of protection was highest for 3−5 days of protection (4.4), followed by 1−2 days (1.9). A product which had 6−24 hours of protection was negligible (−0.4) when making trade‐offs, whereas a product having less than 6 hours of protection (−5.9) was noted as reducing the overall value of a product. The relative utility related to the product's frequency of use was higher if it could be used weekly (5.3). While the relative utility of a product used every other day was negligible (0.2), the need to use a product daily (−5.5) lowered the overall utility of a product. The availability of an over‐the‐counter option offered greater relative utility (2.9) than a product requiring a prescription.

Based on these data, the preferred combination of attributes (i.e. optimal product bundle) for MSM and transgender people who engage in RAI was an enema used 30 minutes before sex, with 95% effectiveness, offering a 3‐ to 5‐day protection window, used weekly, having no side effects and available as an over‐the‐counter product.

## 4 DISCUSSION

End‐user perspectives are vital to the design and testing of new biomedical HIV prevention products prior to their rollout and scale‐up [7, 26, 27, 28]. The present study aimed to investigate end‐user perspectives on three modalities that could deliver a future RM. We used a conjoint experiment to assess the relative importance of different features of rectal delivery modalities after participants had experienced all three modalities. Participants assigned differential importance to the features included in our conjoint experiment, with effectiveness and side effects emerging as the most salient features, consistent with prior research in this area [7, 20, 29]. Furthermore, participants’ preference shares and relative utility values differed depending on whether they were considering attributes as stand‐alone components within a feature, or if they were having to choose an attribute that would be included with others to create an optimal product bundle. Overall, our findings suggest that a one‐size‐fits‐all approach may not be appropriate when designing and testing non‐gel dosage forms for local biomedical prevention during anal sex. Our study underscores the importance of understanding end‐user perspectives when developing new biomedical prevention products and highlights the need for ongoing research in this area to ensure that emerging HIV prevention products are acceptable to end‐users.

When features were considered in isolation, the enema emerged as the most preferred modality. The endorsement of the enema as the most preferred modality for an RM candidate is in alignment with existing sexual behaviour patterns, as sexual and gender minorities (SGM) report the use of enemas prior to anal sex [13, 23, 30, 31]. Nevertheless, if an enema were not an option, participants were more willing to prefer the insert when compared to the suppository when designing a combination product. While our findings align with the existing limited research indicating that both modalities might be acceptable if an RM was effective to prevent HIV [15], these findings align with prior research noting lower acceptability for a suppository relative to other RM formulations [18, 32, 33] (e.g. gels). Given the costs and resources required to design and scale a Phase III clinical trial with an RM candidate, our findings suggest that prioritizing the development of an RM enema may be more promising than a fast‐dissolving insert or suppository candidate, assuming that all other features are comparable. Additional product properties such as stability/shelf‐life, manufacturability, cost and target population should also be considered.

The findings of this study have important implications for HIV prevention. First, the study identified the most important attributes that influence participants’ choice of a product for HIV prevention, including effectiveness, delivery modality and side effects. These findings suggest that future product development should prioritize these attributes to maximize the acceptability and uptake of HIV prevention products. Second, the study found that participants preferred a combination of attributes that included a product with high effectiveness (95%), a 3‐ to 5‐day protection window, and no side effects, used weekly and available over‐the‐counter. These findings suggest that a product with these characteristics would be most appealing to potential users and could potentially improve the overall uptake of HIV prevention products. Third, the study found that participants’ preferences for a product varied based on factors, such as the duration of protection, timing of use before sex and frequency of use. In other words, the development of an RM product must not only be effective, but it must also be convenient to the end‐user. For example, while participants assigned comparable utility to a product that could be used right before sex or 30 minutes before sex, they were less tolerant of a product where one had to wait over an hour before sex after its use. These findings highlight the importance of tailoring HIV prevention products to meet the diverse needs and preferences of potential users.

This study had several strengths. First, this is the first study to examine SGM’ preferences across three understudied modalities for RM delivery prior to RAS. The use of placebo modalities allowed us to focus on participants’ experiences with the dosage form without the potential confounding of drug‐related effects or interactions. Second, the randomized crossover trial design offered participants an opportunity to use all three modalities prior to completing the conjoint analysis. Their ability to use each product prior to evaluating the features and attributes strengthens the social validity of our findings and circumvents prior critiques regarding the limitations of hypothetical assessment when considering the potential of a future RM product. Third, we recruited and retained a large sample of young SGM participants strengthening the representation of SGM communities across diverse geographies and socio‐political settings.

Several limitations deserve discussion. First, we recruited a convenience sample of SGM individuals who were willing to use each of the study products as required by the protocol. We are unable to assess how the protocol's sample selection criteria may affect the overall generalizability of our findings. Second, we were unable to assess whether participants’ trade‐offs in the conjoint experiment would vary based on other decision‐making criteria. For example, a recent review of PrEP product acceptability [7] found that most decision‐making studies have excluded intuitive and emotional components offered by PrEP products (i.e. feelings regarding the product's behavioural congruence with their lifestyles; e.g. reducing worry about HIV acquisition, improving pleasure and intimacy). Similarly, other unassessed features (e.g. portability; discreteness) not included in the conjoint might also influence product use [34]. Future research integrating these components into decision‐making experiments is warranted, particularly as existing data [35−37] suggest that these components may influence SGMs’ HIV prevention decision‐making prior to sex. Finally, while we did not observe statistically significant differences across regions once we had corrected for Type I error, our findings are constrained by limited sample sizes across regions. Future research examining whether differences in preferences across RM delivery vehicles exist across socio‐cultural contexts and geography is warranted.

## 5 CONCLUSIONS

MTN‐035 participants weighed product features differently, recognizing the potential to create diverse, behaviourally congruent biomedical options that fit the needs of intended end‐users. As advances in biomedical strategies for HIV prevention continue to emerge, efforts to diversify HIV prevention options will strengthen our ability to reduce new HIV infections among SGM, whether some desire systemic modalities (e.g. daily oral PrEP; PrEP injectables) or prefer shorter‐term protection (e.g. RMs). Our findings underscore the need to employ innovative decision‐making analyses to characterize the trade‐offs that SGM populations are willing to make in future prevention products. Rather than a one‐size fits all product, our findings underscore how SGM may be willing to make trade‐offs across product features and attributes which may impact their acceptability of a non‐gel RM product. Future research examining these three rectal dosage forms with promising drug candidates that can optimize an RM prevention product's overall profile is warranted.

## COMPETING INTERESTS

AL has received funding for investigator‐sponsored research projects from Gilead Sciences and Viiv Healthcare. Gilead Sciences has donated study drug to studies led by AL.

## AUTHORS’ CONTRIBUTIONS

JB analysed the data and drafted the initial version of the manuscript. All authors (JB, WL, RT, AL, SC, CH, PG, KH, NK, TP‐P, SJ, EB, JZ, CEJ, GFD and JP) contributed to the design and implementation of the research and provided feedback, reviewed and edited the draft, and approved the final version prior to submission.

## FUNDING

The study was designed and implemented by the Microbicide Trials Network (MTN) funded by the National Institute of Allergy and Infectious Diseases (UM1AI068633, UM1AI068615, UM1AI106707), with co‐funding from the *Eunice Kennedy Shriver* National Institute of Child Health and Human Development and the National Institute of Mental Health, all components of the U.S. National Institutes of Health. CONRAD manufactured and provided the inserts for this study with funding from PEPFAR through a cooperative agreement between the U.S. Agency for International Development (USAID) and Eastern Virginia Medical School (AID‐OAA‐A‐14‐00010).

## DISCLAIMER

The content is solely the responsibility of the authors and does not necessarily represent the official views of the National Institutes of Health or any USG agencies.

## ETHICAL APPROVAL STATEMENT

All procedures performed in studies involving human participants were in accordance with the ethical standards of the institutional and/or national research committee and with the 1964 Helsinki Declaration and its later amendments or comparable ethical standards.

## INFORMED CONSENT

Informed consent was obtained from all individual participants included in the study.

## CLINICAL TRIALS REGISTRATION

NCT03671239

## Data Availability

The data that support the findings of this study are available from the corresponding author upon reasonable request.
